# A Central Nervous System-Dependent Intron-Embedded Gene Encodes a Novel Murine Fyn Binding Protein

**DOI:** 10.1371/journal.pone.0149612

**Published:** 2016-02-22

**Authors:** Noureddine Ben Khalaf, Safa Taha, Moiz Bakhiet, M. Dahmani Fathallah

**Affiliations:** 1 College of Postgraduate Studies, Arabian Gulf University, Manama, Bahrain; 2 HH Al-Jawhara Centre for Molecular Medicine and Inherited Diseases, Manama, Bahrain; Seoul National University, REPUBLIC OF KOREA

## Abstract

The interplay between the nervous and immune systems is gradually being unraveled. We previously reported in the mouse the novel soluble immune system factor ISRAA, whose activation in the spleen is central nervous system-dependent. We also showed that ISRAA plays a role in modulating anti-infection immunity. Herein, we report the genomic description of the *israa* locus, along with some insights into the structure-function relationship of the protein. Our findings revealed that *israa* is nested within intron 6 of the mouse *zmiz1* gene. Protein sequence analysis revealed a typical SH2 binding motif (Y_102_TEV), with Fyn being the most likely binding partner. Docking simulation showed a favorable conformation for the ISRAA-Fyn complex, with a specific binding mode for the binding of the YTEV motif to the SH2 domain. Experimental studies showed that in vitro, recombinant ISRAA is phosphorylated by Fyn at tyrosine 102. Cell transfection and pull-down experiments revealed Fyn as a binding partner of ISRAA in the EL4 mouse T-cell line. Indeed, we demonstrated that ISRAA downregulates T-cell activation and the phosphorylation of an activation tyrosine (Y_416_) of Src-family kinases in mouse splenocytes. Our observations highlight ISRAA as a novel Fyn binding protein that is likely to be involved in a signaling pathway driven by the nervous system.

## Introduction

The nervous and immune systems have long been considered to be separate compartments that fulfill different functions, but recent studies showed the existence of a mutual interaction between the two systems in both physiological and pathological conditions. Indeed, cytokines released by immune cells are able to signal to the brain the occurrence of infections [1, 2] by directly accessing the CNS [3] or by stimulating afferent neurons [4]. In response to environmental changes, the brain can modulate immune system functions and maintain homeostasis through a network of neurotransmitters and specific receptors expressed on immune cells [5]. The modulation of immune functions by the CNS occurs in different ways: the autonomic nervous system, the catecholaminergic pathway, or neuropeptide and hormone release [6]. In the spleen, brain immunoregulatory actions can be mediated by the autonomic nervous system through sympathetic and vagus nerve innervations. Vagus nerves excitation induces neuroephinepherine (NE) release from splenic nerves. NE binds to adrenergic receptors on splenic T cells, stimulates acetylcholine production [7] and acts as an immune suppressor. This phenomenon is known as the inflammatory reflex, in which cytokine production is reduced following the binding of acetylcholine to α7 nAChR receptors on macrophages in the spleen red-pulp and marginal zone [8, 9]. These observations pointed toward spleen innervations as a key step in the regulation of immune system activity. As reported by our group in 2008 [10], we used a differential display approach to demonstrate that a novel 2.1 Kb mouse gene (*israa*), which encodes a 125 amino acid protein (ISRAA), is overexpressed in splenocytes less than one minute after *Trypanosoma brucei* parasite inoculation in mice. This rapid induction of *israa* expression in the spleen occurred seconds after the subcutaneous inoculation of the parasite. This induction is significantly inhibited by surgical denervation of the spleen. ISRAA was shown to be secreted by splenocytes and to modulate the immune response in a dose-dependent manner [10]. These observations suggested that *israa* could be involved in the nervous system’s immune-regulatory function. In an attempt to further characterize *israa*, we performed *in silico* structure to function analysis, followed by experimental functional characterization of the protein. The gene (GenBank: EU552928) was mapped to chromosome 14. To investigate the function(s) of this gene, we first analyzed *israa*'s gene structure and genomic environment in the mouse genome. Then, we used a computational approach to gain insights into ISRAA’s structure-function(s) relationship. Protein motif analysis revealed the presence of a typical linear SH2 binding motif exposed on ISRAA's surface. Experimental work provided evidence that ISRAA is likely to be a novel binding protein of Fyn kinase and is likely involved in the regulation of early T-cell activation and signaling.

## Materials and Methods

### Genomic Analysis

Ensembl Blast was used to map the location of *israa* in the mouse genome. The NCBI Blast program was used to perform a similarity search and to investigate expression data. Complete and annotated mRNA sequences of the *zmiz-1* gene in mouse (REFSEQ accession NM_183208.4)) were downloaded in GenBank format [11] from the NCBI database. The Artemis [12] software available from the Wellcome Trust Sanger Institute website was used to visualize and annotate sequences. Regulatory elements were analyzed via the TIS Miner servers.

### Protein Sequence Analysis

Signal peptide, trans membrane topology and protein localization predictions were performed with the SignalP 4.1 server [13], TMHMM server v2.0, SecretomeP [14] and the Phobius server [15] using default parameters for eukaryotic systems. The prediction of putative mitochondrial and ER targeting sequences was performed using the Predotar server, and peroxisomal targeting signal prediction was performed using PTS1 predictor [16]. Leucine-rich nuclear export signal prediction was performed using the NetNES 1.1 server [17]. Putative serine/tyrosine and threonine phosphorylation were predicted using the NetPhos 2.0 server [18], and kinase specificity was predicted using the NetPhosK1.0 server [19]. The conserved domain database (CDD) [20] and SMART [21] servers were used to identify and annotate putative conserved domains. Short protein sequence motifs were analyzed using the Scansite 3.0 server [22] at high stringency.

### Protein Modeling

ISRAA was submitted to the full-chain structure prediction server ROBETTA [23], and four models were generated. The stereochemical quality and accuracy of the predicted models were evaluated with PROCHECK [24] via Ramachandran plot analysis [25]. The best model was selected based on the overall G-factor and the number of residues in the core, allowed, generously allowed and disallowed regions. The selected model was further analyzed using Verify 3D [26]. ProSA [27] was used to display Z-scores. Models were visualized manually using Pymol [28].

### Protein-Protein Docking

Protein models of ISRAA and the crystal structure of the murine Fyn SH2-SH3 domains (PDB id: 3UF4) were refined with the GROMOS 96 implementation of Swiss-PDB Viewer [29]. Protein-protein docking between the Fyn SH2-SH3 domains as a receptor and the ISRAA model as a ligand was performed using the Hex 8.0.0 software [30] in unbound mode. Docking was performed 10 times, and the solutions were generated and clustered. Out of 1,280 clusters, the best conformation was selected manually based on energy and previously described binding modes for SH2 domains. The following parameters were used in the Docking controls of Hex 8.0.0: Correlation type-Shape only; FFT mode-3D; Grid dimension-0.6; Solutions-2,000; Receptor range-180; Step size-7.5; Ligand range-180; Step size-7.5; Twist range-360; Step size-5.5; Distance range-40; Scan step-0.8; Substeps-0; Steric scan-18; and Final search-25. In contrast, the parameters used in clustering controls to obtain the best results are as follows: Max Clusters-500; Sort solutions by-Cluster; Display clusters-Best; Cluster window-200; RMS threshold-3.0; and Bumps threshold-3. Complexes were visualized with Pymol [26].

### Plasmids

The coding sequence for the ISRAA protein was synthesized by GeneCust (GeneCust, Dudelange, Luxemburg) and sub-cloned into the pET-28a+ system (Novagen, Madison, WI, USA) and p-SELECT-zeo (Thermo Fisher Scientific, Waltham, MA, USA) for N-ter 6-His-tagged recombinant protein expression.

### Recombinant Protein Production in *Escherichia coli* and Purification

The recombinant ISRAA protein (rISRAA) and a mutated form (Y102A) were expressed as N-ter 6-His-tagged proteins in a Bl21 (DE3 pLysS) *E*. *coli* strain. Briefly, coding sequences were synthesized by GeneCust (GeneCust, Dudelange, Luxemburg) and cloned into the pET-28a+ system (Novagen, Madison, WI, USA). Competent cells were transformed with recombinant plasmids using the CaCl_2_ method [31]. The selected clones were grown overnight in LB broth at 37°C. Subcultures were grown for 4 h at 37°C then induced for 16 h with 1 mM Isopropyl β-D-1-thiogalactopyranoside (IPTG) at 25°C. Purification of the fusion protein was performed via affinity chromatography using imidazole gradient elution over a Ni-NTA resin according to the manufacturer’s recommendations (GE Healthcare Bio-Sciences, Pittsburgh, PA, USA)). Purity was demonstrated via 15% polyacrylamide gel electrophoresis (PAGE) and silver staining. The selected fractions were desalted via gel filtration chromatography over PD-10 columns according to the manufacturer’s recommendations (GE Healthcare, Buckinghamshire, UK). Endotoxins were removed using the Pierce High-Capacity Endotoxin Removal Spin Column (Thermo Fisher Scientific, MA, USA), according to the manufacturer’s recommendations. Protein concentrations were determined using the Bradford method. Protein purity was assessed using 12% SDS-PAGE and silver staining.

### Tyrosine Kinase Activity Assay

Fyn Tyrosine kinase (TK) activity was determined using the Universal Tyrosine Kinase Activity Kit (Takara Bio, Shiga, Japan) according to the manufacturer’s recommendations. Briefly, recombinant active mouse Fyn (Life Technologies, Carlsbad, CA, USA) was incubated at various concentrations (0.5 to 5 μg/ml) for 20 min at room temperature on a coated universal substrate tyrosine-containing peptide. The reactions were started by the addition of 10 mM ATP and incubated for 30 min at 37°C. The samples were removed, and the wells were blocked for 30 min at 37°C. An anti-phospho-tyrosine antibody (PY-20) coupled to horseradish peroxidase was added, and the plates were incubated for 30 min at 37°C. The antibody was discarded, and the substrate, 3,3'-,5,5'-Tetramethylbenzidin (TMBZ), was added to the wells for 15 min at room temperature. Solution coloration was stopped and measured at 450 nm using a SpectraMax microplate reader (Molecular Devices, Sunnyvale, CA, USA). One unit (U) of enzymatic activity is defined as the amount needed to incorporate 1 pmol of phosphate into the substrate per min. To investigate if rISRAA is a potential substrate of Fyn tyrosine kinase activity, we used a universal kinase activity kit (R&D Systems, Minneapolis, MN, USA). The principle of the assay is to measure the inorganic phosphate that is released from ADP via substrate phosphorylation by the kinase. Different concentrations of mouse Fyn (0.5 and 1.5 μg/ml) were incubated in the presence of 200 μM ATP and 1 μM concentrations of rISRAA and the rISRAA-Y102A mutant for 10 min at room temperature. ADP was used as a positive control, and inorganic phosphate was used for standard curve determination. The reaction was stopped by malachite green. The plates were incubated for 20 min at room temperature. The optical density was measured at 620 nm using a SpectraMax microplate reader (Molecular Devices, Sunnyvale, CA, USA), and the reaction mixtures were analyzed for phospho-tyrosine content via western blotting. For all experiments, each condition was tested in triplicate to ensure the accuracy of the results, and standard deviations were used for error representation.

### Cell Culture and Transient Transfection

The EL4 (*Mus musculus* lymphoma, ATCC id: TIB 39) mouse T-cell line and Expi293F cells (catalog id: A14527, Thermo Fisher Scientific, Waltham, MA, USA) were transfected with the p-SELECT-zeo-rISRAA construct expressing N-terminal 6His-tagged rISRAA or with p-SELECT-zeo-HGh expressing human growth hormone (HGh) as a positive control for expression. p-SELECT-zeo was used for mock transfection. Transfection was performed with the Expifectamine kit (Gibco, Gaithersburg, MD, USA) according to the manufacturer’s protocol. Briefly, the cell lines were maintained in Dulbecco’s modified Eagle’s medium (DMEM) (Sigma-Aldrich, St. Louis, MO, USA) supplemented with 10% fetal bovine serum (Gibco, Gaithersburg, MD, USA), 2 mM glutamine, 0.1 mg/ml of streptomycin and 100 U/ml of penicillin at 37°C with 5% CO_2_. Growth was performed in Expi293F expression medium (Thermo Fisher Scientific, MA, USA) until the cells reached a density of 2.5x10^6^ cells/ml. Viability was confirmed to be 95% using the Trypan Blue dye exclusion method. DNA (30 μg) was mixed with 80 μl of the Expifectamine reagent in an appropriate volume of OptiMEM media and added to the cells. Zeocin was subsequently added for selection at concentrations ranging from 100 to 200 μg/ml. Forty-eight hours post-transfection, the cells were harvested and assayed for recombinant protein expression via western blotting.

### Pull-Down Experiment

Expi293F cells transfected with p-SELECT-zeo-rISRAA were harvested after 48 hours and lysed. Pull-down assays were performed using the Pierce His Protein Interaction Pull-Down Kit (Thermo Fisher Scientific, MA, USA) according to manufacturer’s recommendations. Briefly, equilibrated cobalt resin beads were incubated with Expi293F cell lysates for 30 min on ice. Untransfected cells were used as a negative control. The beads were washed 5 times and stored. A bead fraction was boiled in SDS buffer and then analyzed for rISRAA immobilization via western blotting using a polyclonal rabbit anti-His-Tag antibody at a final dilution of 1:1000 (Cell Signaling Technology, Danvers, MA, USA) and an anti-rISRAA monoclonal antibody used at a final dilution of 1:100 (GenScript, Piscataway Township, NJ, USA). EL4 cell lysates were prepared and incubated with rISRAA immobilized beads for 2 hours with gentle rotation at 4°C. After washing, the protein complexes were eluted from the beads with 300 mM imidazole. The eluted fractions were analyzed for Fyn protein detection via western blotting using a rabbit polyclonal anti-Fyn antibody (Abcam, Cambridge, UK) used at a final concentration of 1:1000.

### Preparation of Splenocytes

This work was approved by the Ethics and Research Committee of the Arabian Gulf University and was carried out in strict accordance with the recommendations in the committee’s guide for the use of laboratory animals. All animal euthanasia was performed via barbiturate overdose (100 mg/Kg) through intravenous injection, and all efforts were made to minimize animal suffering. Eight-week-old female Balb/c mice were used to prepare splenocytes. The spleens were dissected, and splenocytes were prepared by forcing the spleens through a stainless steel mesh. The freed cells were washed once in PBS. Hemolysis of erythrocytes in the cell pellets was performed by adding 2 ml of cold water for 30 s, followed by the addition of 1 ml of Phosphate-buffered saline (PBS buffer). The cells were washed twice in RPMI medium supplemented with L-glutamine, penicillin and streptomycin (Sigma-Aldrich, MO, USA) and then re-suspended in the same medium supplemented with 10% Fetal Calf Serum (FCS) (Life Technologies, CA, USA) to obtain a concentration of 2x10^6^ cells/ml.

### Anti-CD3 Cell Activation Assay

The 3-(4,5-Dimethylthiazol-2-yl)-2,5-Diphenyltetrazolium Bromide (MTT) Cell Proliferation Assay (ATCC, Manassas, VA, USA) was used to measure cellular activation by anti-CD3. The plates were coated overnight at 4°C with anti-mouse-CD3 (BD Biosciences, San Jose, CA, USA) at a concentration of 5 μg/ml in PBS. The control wells were coated with PBS alone. The cell suspension was harvested by centrifugation and re-suspended at 2x10^6^ cells/ml in complete RPMI medium supplemented with 10% FCS (Sigma-Aldrich, St. Louis, MO, USA). One hundred microliters of the cell suspension were plated in each well. Control wells with culture medium alone provided the blanks for absorbance readings. An equal volume of medium containing rISRAA at a final concentration of 1 μM was added to the test wells. Medium alone was added to the control wells. All conditions were tested in triplicate. The plates were incubated at 37°C in 5% CO_2_ for 24 hours. Later, 10 μl of 5 mg/ml MTT reagent was added to each well, including the controls, and incubated for 4 hours at 37°C. The plates were centrifuged, and the medium was removed from the wells. Then, 100 μl of DMSO were added to all of the wells. The plates were covered with aluminum foil, agitated on an orbital shaker for 15 min and incubated for 4 h at 37°C to completely dissolve the Formazan crystals. Finally, the absorbance was recorded at 570 nm, with 620 nm as a reference wavelength using a SpectraMax microplate reader (Molecular Devices, Sunnyvale, CA, USA), and the stimulation index was calculated as follows:

$$Stimulation\;Index=\frac{ODexperiment-ODblank}{ODcontrol-ODblank}$$

### SFK Phosphorylation

The splenocyte suspension was harvested by centrifugation and re-suspended at 2x10^6^ cells/ml in complete RPMI medium supplemented with 10% FCS, and 400 μl of the cell suspension were plated in 12-well plates. An equal volume of medium containing recombinant rISRAA was added to the test wells at a final concentration of 1 μM, and cells alone served as a negative control. After different stimulation times (1, 5, 10, 15 and 30 minutes), the cells were harvested at 10,000g for 15 min, washed in cold 1x PBS and re-suspended in a convenient volume of RIPA buffer (Cell Signaling Technology, Danvers, MA, USA) supplemented with phosphatase and a protease inhibitor cocktail for cell lysis. The cell lysate was clarified by centrifugation at 12,000g for 30 min at 4°C. The soluble protein concentration was determined using the BCA Protein Assay Kit (Thermo Fisher Scientific, Waltham, MA). For western blotting analysis, equal amounts of total protein were resolved via 12% SDS-PAGE. The proteins were transferred to nitrocellulose membranes, blocked in PBS containing 5% nonfat dry milk and 0.1% Tween-20, and then incubated overnight at 4°C with the anti-phospho-Src family (Tyr 416) (Cell Signaling Technology, Danvers, MA, USA) and anti-actin (BD Biosciences, San Jose, CA, USA) antibodies. The antibodies were used at a dilution of 1:1,000 for SFK phosphorylation. The respective HRP-conjugated secondary antibodies (Cell Signaling Technology, Danvers, MA, USA) were used at a dilution of 1:1,000. The bands were detected using an enhanced chemiluminescence kit (GE Healthcare, Buckinghamshire, UK), and the membranes were scanned using an image analyzer (LAS-1000, Fuji Film, Tokyo, Japan).

### Statistical Analysis

The two-tailed t-test for independent samples was used to calculate the level of significance (*p<0.05, **p<0.01, and ***p<0.001) for all experiments. All calculations were performed using SPSS statistics software version 19 (www.ibm.com).

## Results

### *Israa* Is an Intron-Embedded Gene

**Fig 1:**
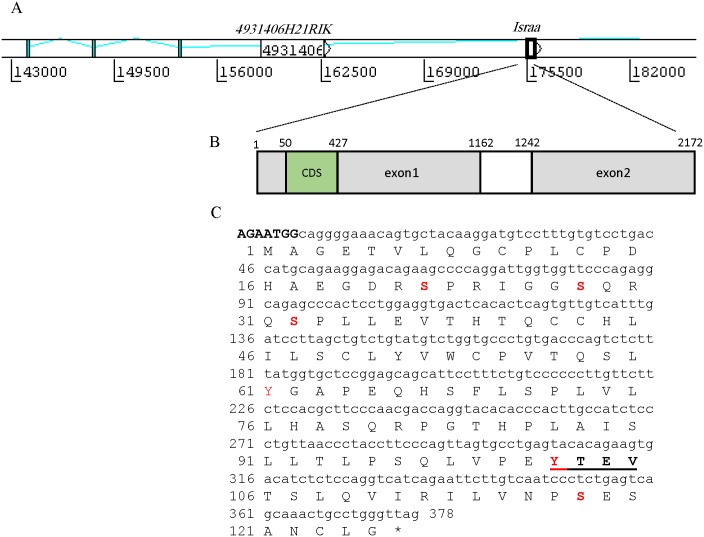
Structural analysis of the *Israa* gene. (A) *israa* is embedded in intron 6 of the *zmiz1* gene on mouse chromosome 14. The 4931406H21RIK gene is located 13 Kb upstream of *israa*’s CDS. (B) The *israa* gene is transcribed in two exons; the CDS is present in exon 1. (C) Nucleic and amino acid sequences of *israa*. The predicted Kozak consensus region is represented in uppercase letters. Predicted phosphorylation sites are highlighted in red. The motif predicted to interact with the SH2 domain of Fyn is represented in underlined bold font.

Ensembl Blast analysis showed that *israa* is a 2,093 bp gene embedded within intron 6 of the *zmiz1* gene on mouse chromosome 14 (Fig 1A). *zmiz1* encodes a 1,072 amino acid protein: the zinc finger, MIZ-type containing 1 protein (NP_899031.2), which is also known as zimp10 and is located in region 14qa3 of mouse chromosome 14. The *zmiz1* gene is composed of 24 exons and is transcribed into a 207,563 bp pre-mRNA. *israa* is located approximately 22 kb downstream of *zmiz1* exon 6 and 30 kb upstream of exon 7. NCBI blast analysis showed that the *israa* coding DNA sequence (CDS) (378 bp) is present with 100% identity in two mouse cDNA libraries of ESTs (dbEST ids: 2406142 and 8663286). An exhaustive Blast search revealed no orthologs for the *israa* gene, and an analysis of *israa*’s transcript showed that it consists of two exons. The junction between the exons is located at base 1,162, and the DNA segment corresponds to a fragment 79 bp in length. An analysis of this junction region revealed the presence of a conventional splicing acceptor sequence but no conventional donor site. *israa* encodes a 125 residue protein (GenBank: ACB55610.1). Both the ATG initiation and STOP codons are located in exon 1 (Fig 1B). Prediction of the Kozak consensus region using the TIS Miner server showed a significant score (0.786) for the sequence AgaATGG at the translational start site of the *israa* CDS, which indicates that the *israa* mRNA can be translated in mammalian cells. In addition, another nested gene (4931406H21RIK) is located 12,886 bp upstream of *israa* (GenBank: NR_033492.1), with an open reading frame (ORF) of 3,858 bp (Fig 1A). The RIK gene is constituted by a unique exon and encodes a putative protein of 145 residues in length (GenBank: AAI47213). No ortholog for this gene has been found in the human genome, and no function has been reported for this gene. Expression data obtained via in situ RNA analysis showed tissue-specific gene transcription in the nervous system in mouse embryos [32]. The 4931406H21RIK gene product showed no significant similarity with other proteins or with known conserved protein motifs and functional domains.

### ISRAA Protein Analysis and Modeling

**Fig 2:**
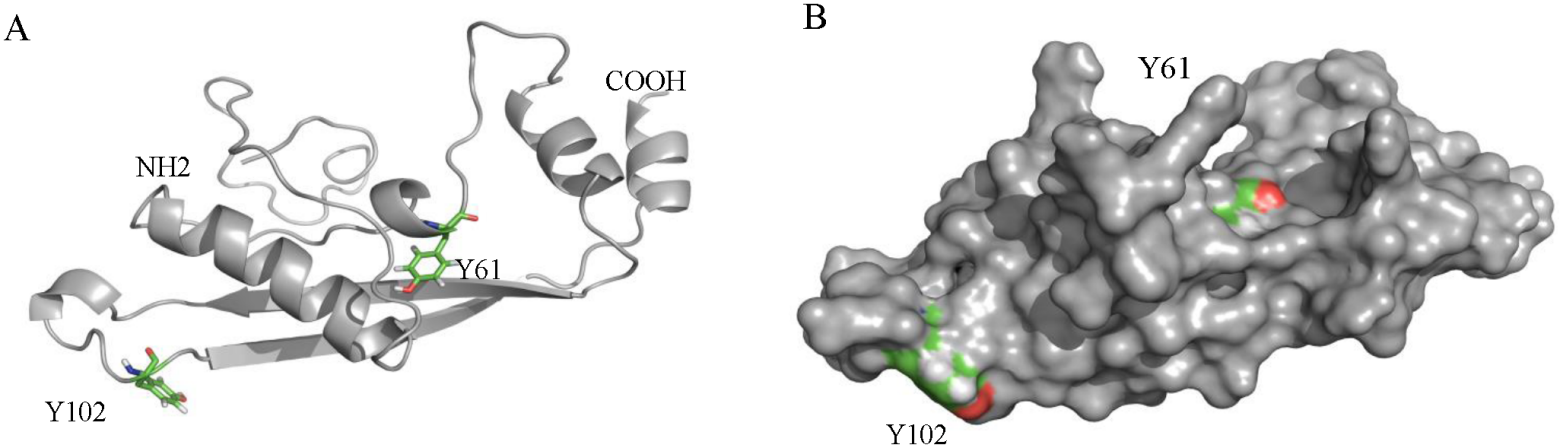
*De novo* model of ISRAA. (A) Cartoon representation of the ISRAA model showing folding into two antiparallel β-sheets, three helices and three major loops; this model does not display any common protein domain folds. (B) Surface representation of the predicted ISRAA model. Tyrosine residues 61 and 102 are colored by elements. Y_102_, which is part of the predicted SH2 binding motif, is exposed on the protein surface, whereas Y_61_ is shown to be partially exposed.

ISRAA is formed by 125 amino acids and has a predicted molecular weight of 13.5 KDa, with a pHi of 5.77. Using the NetPhos 2.0 server, we identified several residues that are potential phosphorylation sites: serine residues 22, 28, 32, and 118 and tyrosine residues 61 and 102 (Fig 1C). Kinase specificity analysis revealed Src-family kinases (SFKs) as the putative specific kinases for Tyr 61 and 102, with a score of 0.5 (Fig 1C). A potential palmitoylation site is located at cysteine 13, and an O-ß-GlcNAc attachment site is located at threonine 57. In addition, ISRAA is predicted to be secreted in an unconventional manner (secretome 2.0 score of 0.931). No potential protein trafficking motifs were identified using different prediction programs, except for a potential leucine-rich nuclear export signal at positions 87–97 (L A I S L L T L P S Q L), with a score slightly superior to the threshold defined by the NetNES server. This signal could be involved in protein export from the nucleus to the cytoplasm. No protein similar to ISRAA was found using multiple blast searches. A comprehensive conserved domain search showed no common conserved functional domains in the protein. Meanwhile, the short protein sequence motif search revealed the presence of a linear “SH2 binding motif” located at tyrosine residue 102 (YTEV), with the lowest score of 0.304 corresponding to Fyn as the best binding candidate (Fig 1C). This motif allows the docking of ISRA into the Fyn-SH2 domain via phospho-tyrosine (p-Tyr) recognition. The ISRAA sequence showed no significant similarity with Protein Data Bank entries. To further characterize the protein structure, we built a de novo model using the ROBETTA full-chain structure prediction server [24]. The best model (Fig 2A) was selected based on the overall G-factor and the number of residues in the core, allowed, generously allowed and disallowed regions. The model showed an overall G-factor of -0.11 and a Z-factor of -3.21. Verify3D analysis showed an overall score of 0.28, indicating a satisfactory model. The folding of the protein is shown in Fig 2A. The model shows favorable surface exposure of the Y_102_ residue and partial surface exposure of the Y_61_ residue (Fig 2B).

### Protein-Protein Docking Simulation

**Fig 3:**
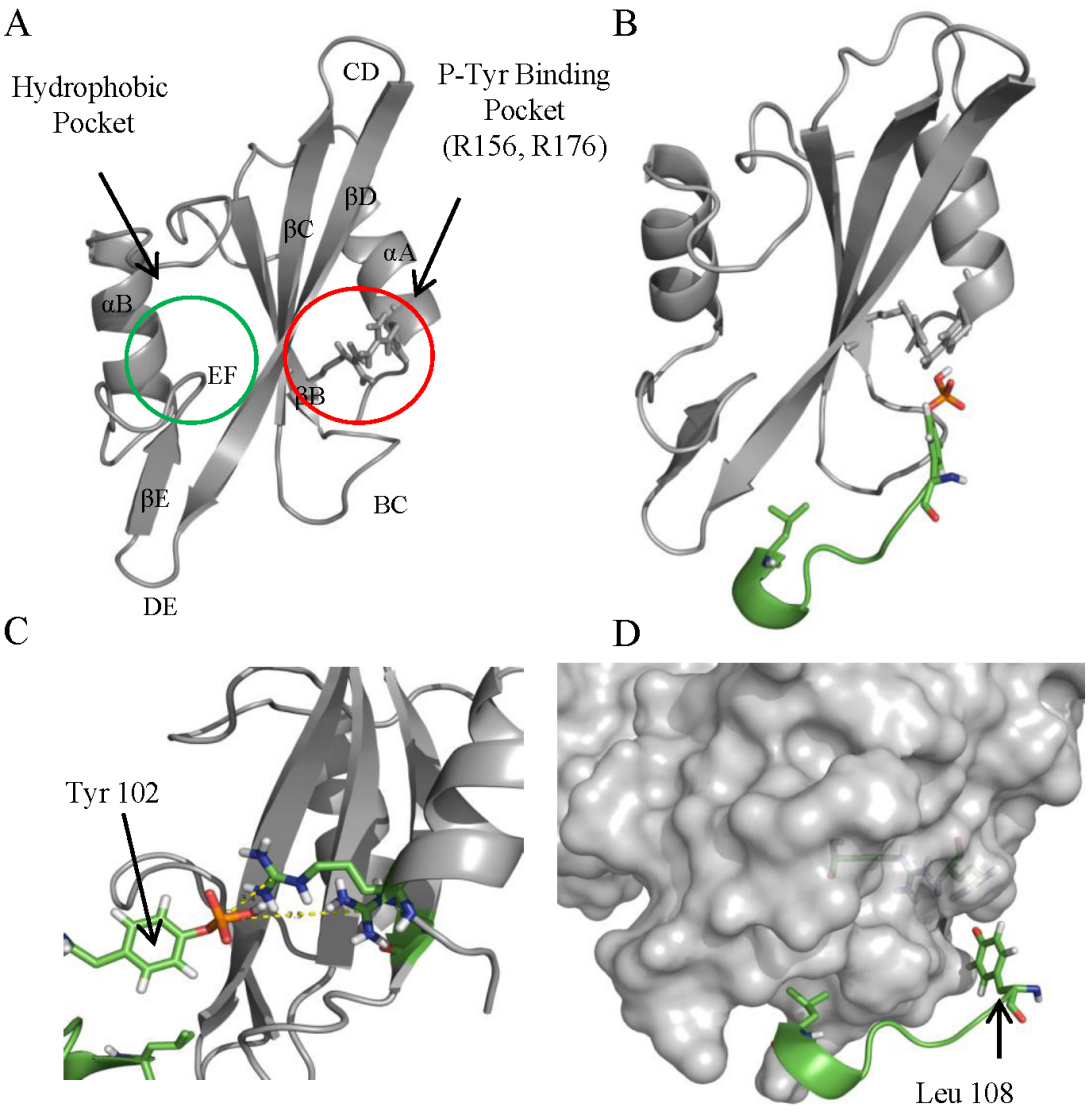
ISRAA-Fyn docking prediction. (A) SH2 domain structure. P-Tyr-containing peptides have two docking sites in the Fyn SH2 domain: a phospho-Tyr binding pocket located between the central anti-parallel βC-sheet and the α-helix αA and a hydrophobic binding pocket near the EF loop. Computational docking of the ISRAA model with the Fyn SH2 domain shows specific binding of Y_102_ to the p-Tyr binding pocket (B), where Y_102_ is located 3.5 A from R_156_ (C). An unconventional binding mode is observed for L_108_ (pTyr+6), which is located in a binding pocket between the βD-sheet and the BC loop (D) that is different from the classic pocket. In the entire figure, the Fyn-SH2 domain is represented in gray, and the ISRAA peptide (Y_102_TEVSTL) is represented in green.

Protein-protein docking between the refined model of the mouse FYN SH2-SH3 domain structure (PDB id: 3UF4) (receptor) and the ISRAA model (ligand) was performed using the Hex 8.0.0 software in unbound mode, as described in the Methods section. Docking was performed 10 times, and the best solutions were selected based on previously described Fyn-SH2 interaction modes with phosphopeptides [33]; R_156_ and/or R_176_ were shown to bind p-Tyr residue ligands, and a hydrophobic pocket of the SH2 domain provides specificity for a hydrophobic residue in the C-terminal position of the pTyr-containing peptide (Fig 3A). Selected solutions satisfying these conditions were shown to be clustered in a single conformation (Fig 3B). The best solution was chosen based on free energy and showed a total energy of -30 KJ/mol, indicating satisfactory stability. Interaction analysis showed a conventional binding mode for the ISRAA Y_102_-containing peptide and the Fyn SH2 domain (Fig 3C and 3D), which will be discussed later.

### ISRAA Is a Substrate of Fyn

**Fig 4:**
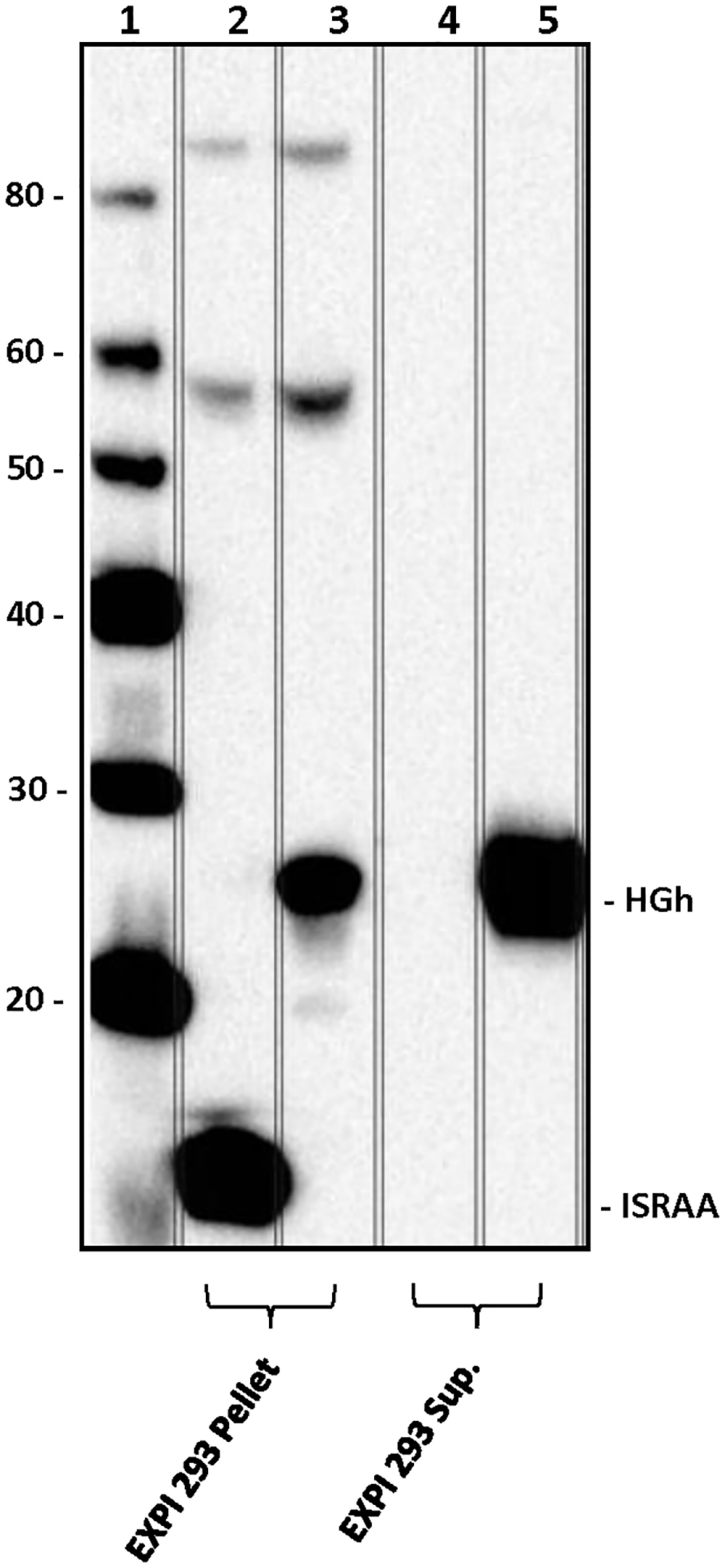
Cell transfection and rISRAA expression. Expi293F cells were transfected with p-SELECT-zeo-ISRAA and selected with zocin, and p-SELECT-zeo-HGh was used as a positive control for expression. After 48 hours of transfection, the cell lysates and supernatants were analyzed via western blotting with an anti-His6 antibody. HGh was strongly expressed by Expi293F cells in both cell pellets (lane 3) and supernatants (lane 5). Expi293F cells strongly expressed rISRAA in an intracellular form at the expected molecular weight (15 KDa) (lane 2), and no rISRAA was detected in the supernatants (lane 4).

**Fig 5:**
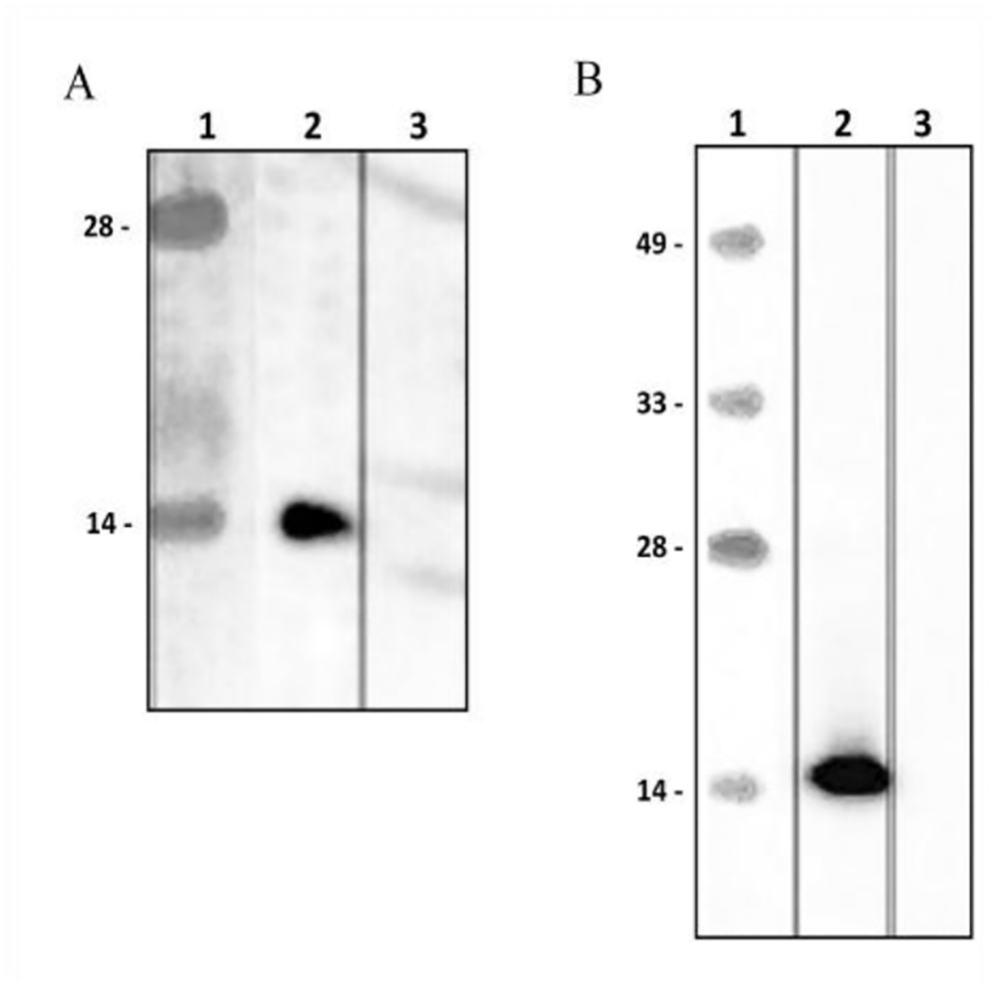
Pull-down of rISRAA. rISRAA was pulled down from Expi293F lysates after transfection using cobalt beadsExpi293F. The beads were boiled and analyzed for rISRAA binding via western blotting with (A) an anti-His antibody (lane 2) and (B) a monoclonal anti-rISRAA antibody (lane 2) (Genescript). Both antibodies detected a 15 KDa protein, which was shown to bind and be pulled down in satisfactory amounts. No cross-reaction with beads incubated with mock-transfected Expi293F lysates was observed for either antibody Expi293F(lane 3 in A and B).

**Fig 6:**
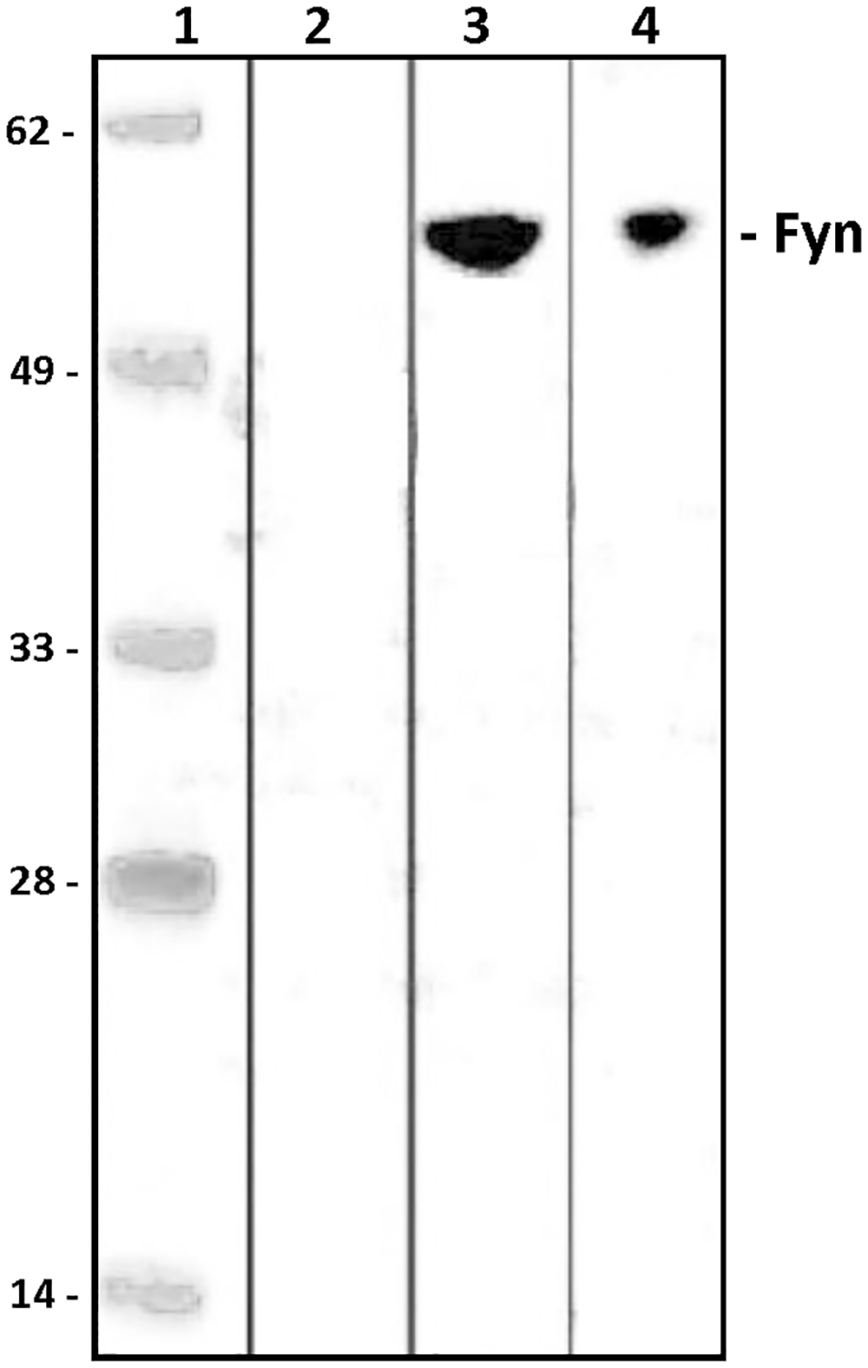
Pull-down assay shows that ISRAA binds to Fyn. Bead-immobilized rISRAA pulled down from Expi293F lysates was used as bait for EL4 cell lysates. Western blot analysis using a monoclonal anti-Fyn antibody did not reveal any detectable Fyn protein in EL4 lysates incubated with beads alone (lane 2). The Fyn protein (59 KDa) was specifically detected in EL4 whole cell lysates (lane 3) and pulled down specifically with rISRAA immobilized beads (lane 4).

To investigate the *in vitro* interaction of the Fyn protein with ISRAA, we transfected EL4 cells, a mouse T-cell line, which failed to over-express the rISRAA protein to a level allowing purification for subsequent use in interaction studies (data not shown). We then over-expressed rISRAA fused to an N-terminal His-tag in Expi293F cells (Fig 4). The recombinant protein was shown to be pulled down by cobalt beads and specifically detected with both an anti-His antibody and a monoclonal anti-ISRAA antibody generated by GenScript (GenScript, NJ, USA) (Fig 5). The rISRAA protein was then immobilized on beads and used as bait for EL4 cell lysates. Western blotting analysis of protein complexes revealed Fyn as prey, showing a specific interaction with the rISRAA protein (Fig 6).

**Fig 7:**
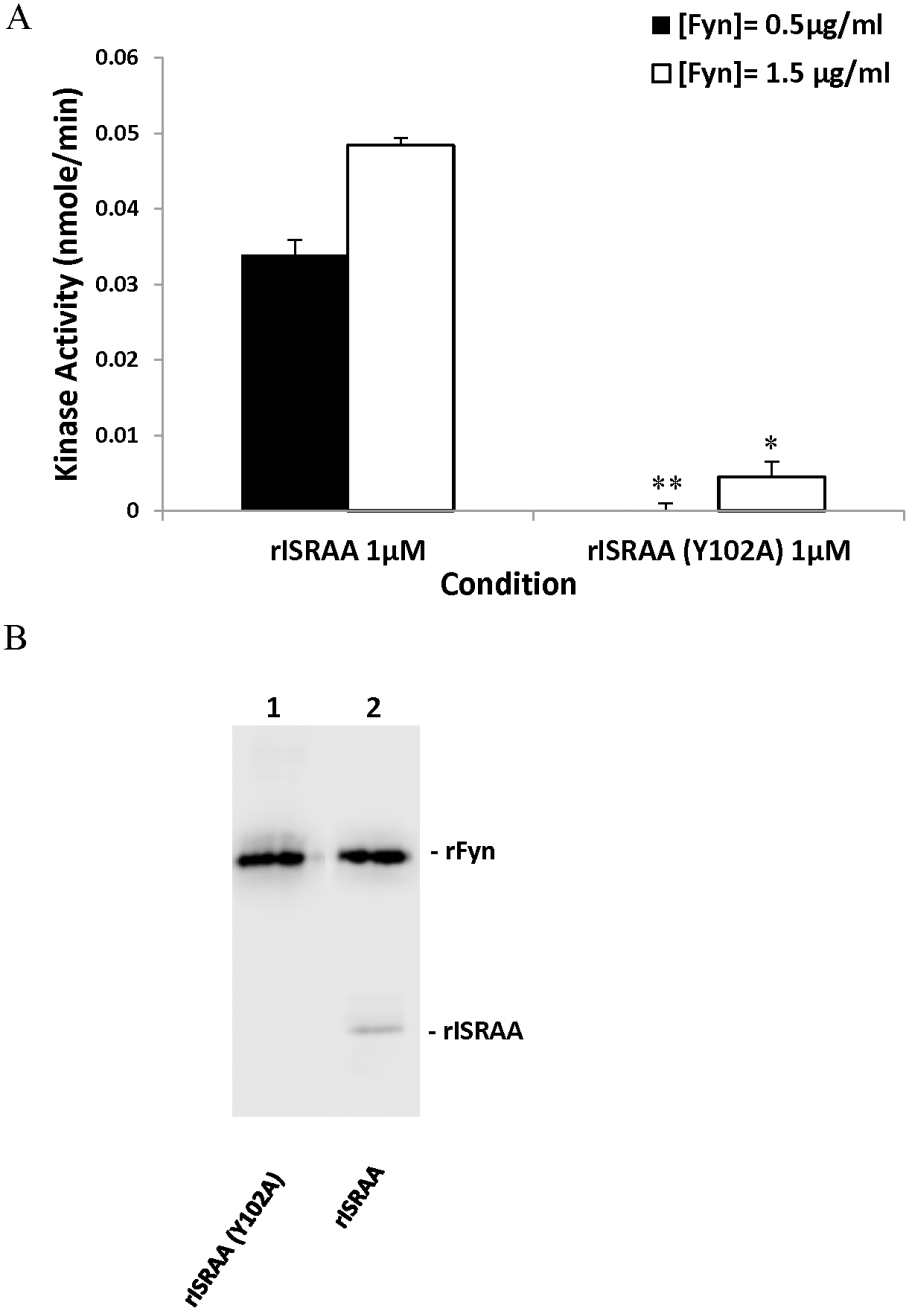
Fyn phosphorylates ISRAA. (A) Direct phosphorylation of ISRAA by the Fyn protein was assayed using different concentrations of mouse Fyn in the presence of 1 μM concentrations of rISRAA and the rISRAA-Y102A mutant. The mutation of the Y_102_ residue significantly affects the kinase activity of Fyn at concentrations of 0.5 μg/ml (**p<0.001) and 1.5 μg/ml (*p<0.01). (B) The kinase reaction mixtures were analyzed via western blotting using an anti-phosphotyrosine antibody (PY-20); lane 1: recombinant Fyn + rISRAA-Y102A, lane 2: recombinant Fyn + rISRAA. Only rISRAA shows phosphorylated tyrosine content.

To investigate the nature of the ISRAA-Fyn interaction, we performed a tyrosine kinase assay of the recombinant Fyn protein to determine its specific activity. The protein showed a significant specific tyrosine kinase activity of 7.5x10^3^ U/mg. We then measured the total Fyn activity in the presence of rISRAA and rISRAA-Y102A as substrates. Fig 7A shows the concentration-dependent enzymatic activity of Fyn (0.03 to 0.05 nmol/min) in the presence of 1 μM rISRAA, while no significant activity was shown when the rISRAA-Y102A mutant was used as a substrate. This experiment shows that rISRAA is a substrate for Fyn, primarily through its Y_102_ residue. The mutation of that residue to alanine abolishes the kinase activity of Fyn on rISRAA. Western blotting analysis of the reaction mixture using an anti-p-tyrosine (PY-20) antibody before the addition of malachite green (Fig 7B) confirmed the p-Tyr content of recombinant rISRAA. In contrast, no significant tyrosine phosphorylation was detected in the rISRAA-Y102A mutant.

### ISRAA Downregulates Early T-cell Activation and SFK Activation

**Fig 8:**
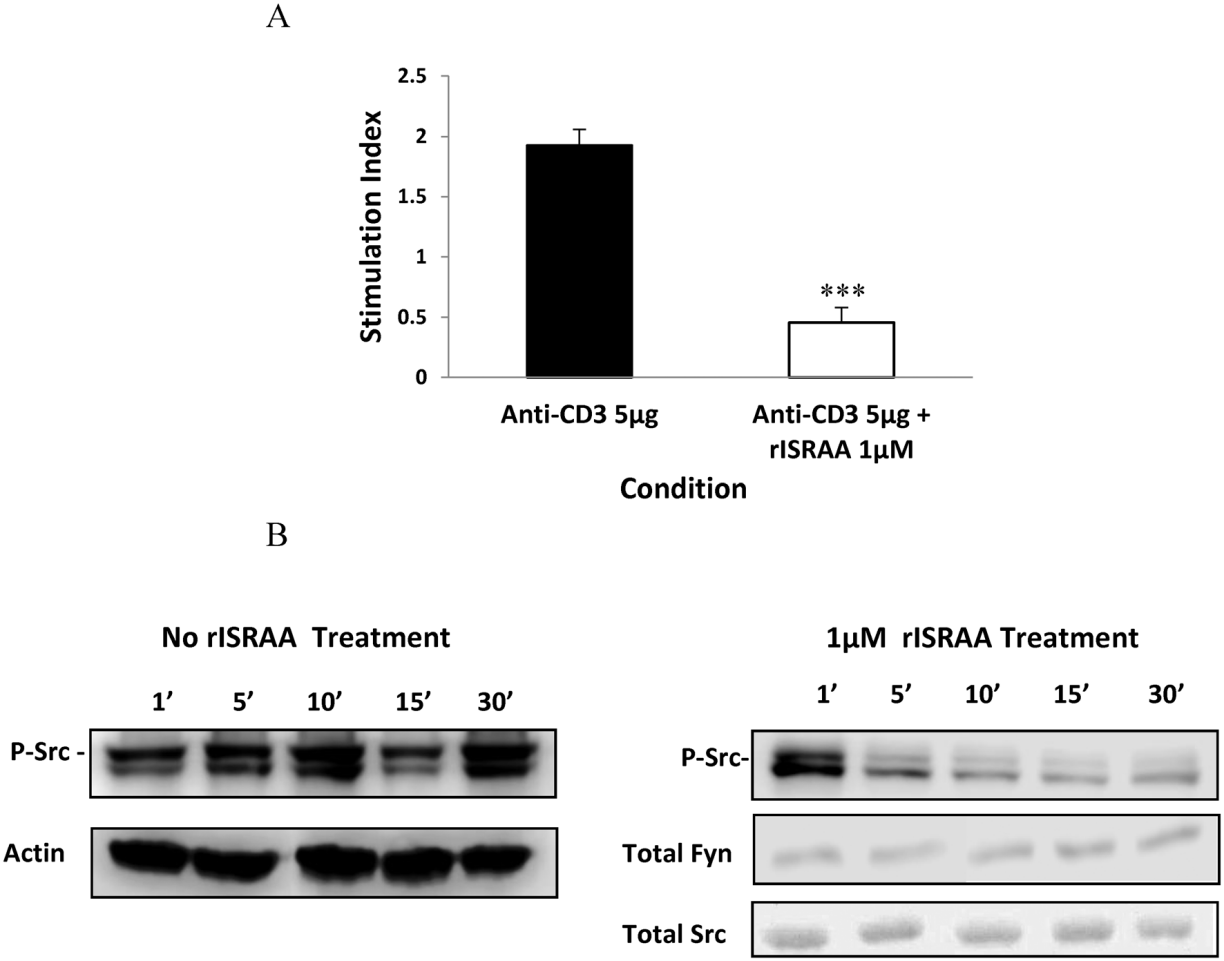
rISRAA affects early cell activation. (A) MTT Cell Activation Assay; T-cell activation by anti-CD3 in the presence and absence of 1 μM soluble rISRAA was measured after 24 h of stimulation of mouse splenocytes. ISRAA significantly downregulates early T-cell activation in comparison to anti-CD3 treatment alone (***p< 0.0001). (B) Western blotting analysis of time-dependent Tyr 416 phosphorylation in Src-family kinases. Treatment of splenocytes with 1 μM rISRAA clearly downregulates the phosphorylation of an activation tyrosine in the SFK members Src (59 KDa) and Fyn (59 KDa). Total Fyn and Src analysis showed equivalent amounts of the proteins in all samples. Untreated cells show equivalent amounts of the actin protein and stable Tyr-416 phosphorylation at all time points.

The MTT cell activation assay was used to measure T-cell activation by anti-CD3 in the presence and absence of 1 μM rISRAA. After 24 h of incubating splenocytes with 5 μg/ml of anti-CD3, the stimulation index was calculated. The positive control (no rISRAA) showed a stimulation index of 2 compared to non-stimulated cells (Fig 8A). Splenocytes incubated with 1 μM rISRAA downregulated T-cell activation (SI < 1) (Fig 8A). Splenocytes treated with 1 μM rISRAA at various times (1, 5, 10, 15 and 30 minutes) and probed using an anti SFK p-Y416 antibody showed a time-dependent dephosphorylation of the SFK members Fyn and Src, as shown by specific protein molecular weights (59 KDa), in comparison to untreated cells (Fig 8B). An analysis of the total actin, Src and Fyn proteins in the cell lysates showed the presence of equivalent amounts of proteins.

## Discussion

*Israa* has been identified in mice and described as a novel immune system modulator whose transcription is activated *in vivo* in a manner dependent on intact splenic innervations. Interestingly, we found that the gene that encodes ISRAA is an intron-embedded gene in intron 6 of the mouse *zmiz1* gene on chromosome 14. This intron also contains another gene, 4931406H21RIK, whose function is also unknown. The CDS of *israa* matched two EST libraries with 100% identity, showing that this ORF is transcribed into mRNA in the same sense as the *zmiz1* transcript. The observation that *israa* contains a Kozak consensus region argues in favor of its translation. These features, along with the original observation of Bakhiet et al. [10], confirm that *israa* is an active mouse gene. However, the mechanism of *israa* transcription and mRNA maturation must be investigated further because no typical splicing features were found in the gene. More intriguing is the fact that we found no orthologs of *israa* in lower vertebrates or primates. Meanwhile, the analysis of the ISRAA protein revealed an extra-cytoplasmic predicted location, although no common signal peptide was identified. The presence of a putative palmitoylation site at Cys13 in the N-terminal part of the protein could indicate potential lipid-raft localization of the protein, therefore strengthening the possibility that ISRAA is released effectively [34]. Indeed, short sequence motif analysis showed the presence of a short linear motif (Y_102_TEV) located at Tyr102 that could bind to SH2 domains, with the best score matching the Fyn SH2 domain, which is prone to phosphorylation by an SFK. In addition, a *de novo* model of ISRAA showed that the Y_102_TEV motif is well exposed on the protein surface, which facilitates its involvement in protein-protein interactions.

Fyn is a member of the SFKs, which are involved in essential signaling pathways, such as cell growth, survival, migration, motility and adhesion [35]. Fyn is localized primarily to the cytoplasmic leaflet of the plasma membrane [35]. Two different isoforms of Fyn are expressed in mice; isoform 1 or B is expressed primarily in the brain, and isoform 2 or T is strongly expressed in T-cells [36]. Fyn plays a central role in immune system signaling and early T-cell activation signaling by phosphorylating T-cell receptor ITAMs in the CD3 and ζ chains. This phosphorylation allows the recruitment of ZAP-70, which activates T-cell adaptor proteins, such as SLP-76 and LAT, leading to secondary messenger generation and the T-cell activation cascade [37]. The protein shares a common multi-domain architecture with other SFKs, including an SH_1_ kinase domain, SH2 and SH3 binding domains, and a C-terminal negative regulatory domain [33]. The SH2 domain is important for directing specific protein-protein interactions that are necessary for substrate specificity, activity regulation, and protein recruitment. Hence, identifying the proteins that bind to this specific domain could help to elucidate the mechanism by which Fyn-related pathways are regulated. Docking simulation of ISRAA within Fyn’s SH2 domain showed a favorable binding mode in which the Y_102_ residue is located, as expected, in the pTyr-binding cavity between the central anti-parallel βC-sheet and the α-helix αA. When phosphorylated, the phosphate group is 3.5 A from R_156_, which allows H-bond formation. The C-terminal residues of the YTEV motif were expected to be located in the hydrophobic binding pocket near the EF loop; however, the analysis showed a different conformation in which Leu108 is located in a binding pocket between the βD-sheet and the BC loop. In fact, previous studies showed that the binding mode of the p-Tyr-containing motif to the SH2 domain depends strongly on its conformation; a 2010 study showed different binding modes of the same p-Tyr peptides to the Src-SH2 domain depending on whether the peptides adopt an extended or helical conformation [38]. Indeed, a major structural characterization of SH2 binding modes was carried out using short linear peptides and failed to provide a complete picture. Hence, further investigations of the binding mode of ISRAA to the Fyn-SH2 domain using mutagenesis and structural characterization could reveal new insights into p-Tyr recognition and binding regulation.

Cell transfection, pull-down experiments and tyrosine kinase assays provided evidence that cellular Fyn binds to the rISRAA protein and that Fyn is able to phosphorylate rISRAA in vitro. Indeed, ISRAA’s Y_102_ residue appears to play a major role in the ISRAA-Fyn interaction and ISRAA phosphorylation. This finding could indicate a complex mode of binding of ISRAA to Fyn, which is known as “processive phosphorylation” [39]. In this process, the kinase domain of a kinase phosphorylates a substrate at the tyrosine site, allowing it to bind to the SH2 domain, leading to further phosphorylation of other tyrosine residues in the substrate or another protein bound to the substrate. In the case of ISRAA, we propose that upon phosphorylation by Fyn, the Y_102_TEV motif binds to the SH2 domain of Fyn, which exposes another tyrosine (e.g. Y_61_) for phosphorylation by the kinase domain of Fyn and leads to the recruitment of other substrates or effectors to the complex. In this case, ISRAA could constitute a new adaptor protein that binds to Fyn and may be involved in the Fyn-dependent signaling pathway. In previous studies, similar approaches succeeded in identifying novel signaling proteins that are involved in Src-family signaling [40].

ISRAA was previously described as a secreted factor that promotes the reactivation and proliferation of *Trypanosome*-induced immunosuppressed splenocytes following rISRAA treatment (10); however, that study was performed using a mixed population of immune cells without targeting a specific cell population. Hence, that study could not indicate a specific role for the ISRAA protein in a specific immune cell population. In an attempt to understand the role of ISRAA in the early T-cell activation process due to its binding with Fyn, we chose to study the effect of rISRAA on T-cell activation induced by anti-CD3. We have shown that CD3-induced activation is down regulated by soluble rISRAA treatment and that this effect is due in part to the dephosphorylation of the activation tyrosine of the SFK members Fyn and Src (p-Src family Tyr-416) in the early minutes after cell treatment with the rISRAA protein. Interestingly, in contrast to the findings reported in 2008 [10], this study shows that ISRAA appears to have a negative effect on early T-cell activation by triggering the dephosphorylation of SFKs at Y416.

Taken together, the evidence presented in this report strongly suggests that *israa* is the first described intron-embedded gene to encode a Fyn binding protein that could be involved in the control of immune homeostasis by the nervous system. Although no *israa* ortholog was found in the human genome, a comparative genomics study revealed several ORFs within the intron 6 region of the human *zmiz*1 homolog that could represent candidates with a similar function. A comprehensive study of the ISRAA signaling pathway and the identification of partner proteins and the mechanisms by which *israa* is regulated could provide an improved understanding of the control of immune function by the nervous system.
